# Conformal thyroidectomy is a feasible option in papillary thyroid microcarcinoma: a retrospective cohort study with 10-year follow-up results

**DOI:** 10.1007/s00423-024-03333-9

**Published:** 2024-05-07

**Authors:** Chen Li, Jian Cao, Guo-Shuai Chen, Xiao-Dong Yang, Ke-Wei Jiang, Ying-Jiang Ye

**Affiliations:** Department of General Surgery, Laboratory of Surgical Oncology, Peking University People’s Hospital, Beijing, 100044 China

**Keywords:** Thyroid cancer, Papillary thyroid microcarcinoma, Conformal thyroidectomy, Prognosis, Retrospective study

## Abstract

**Background:**

In recent years, there has been an increasing prevalence of patients with papillary thyroid microcarcinoma (PTMC) without lymph node involvement in medical centers worldwide. For patients who are unable to undergo active surveillance (AS) and are afraid of postoperative complications, conformal thyroidectomy may be a suitable option to ensure both preservation of function and complete removal of the tumor.

**Methods:**

The patients in the cohort during 2010 to 2015 were retrospectively enrolled strictly following the inclusion and exclusion criteria. The observation and control groups were defined based on the surgical approach, with patients in the observation group undergoing conformal thyroidectomy and patients in the control group undergoing lobectomy. Event-free survival (EFS), the interval from initial surgery to the detection of recurrent or metastatic disease, was defined as the primary observation endpoint.

**Results:**

A total of 319 patients were included in the study, with 124 patients undergoing conformal thyroidectomy and 195 patients undergoing lobectomy. When compared to lobectomy, conformal thyroidectomy demonstrated reduced hospital stays, shorter operative times, and lower rates of vocal cord paralysis and hypoparathyroidism. Furthermore, the mean bleeding volume during the operation and the rate of permanent hypothyroidism were also lower in the conformal thyroidectomy group than in the lobectomy group. However, there was no statistically significant difference observed in the 5- and 10-year EFS between the two groups.

**Conclusions:**

Conformal thyroidectomy had advantages in perioperative management and short-term complication rates, with an EFS that was not inferior to that of lobectomy. Thus, conformal thyroidectomy is a feasible option for low-risk PTMC patients.

**Supplementary information:**

The online version contains supplementary material available at 10.1007/s00423-024-03333-9.

## Background

Papillary thyroid microcarcinoma (PTMC) is defined by the WHO as papillary thyroid cancer with a maximum diameter of 1.0 cm [1]. The incidence of PTMC has increased rapidly and constitutes more than 50% of new thyroid cancer cases [2–4], gradually becoming the main disease component of patients in thyroid medical centers, especially PTMC with no concerning lymph nodes.

Active surveillance (AS) and surgery are two selective choices for PTMC patients without concern for lymph nodes. Referring to guidelines published by the American Thyroid Association (ATA) [5], National Comprehensive Cancer Network (NCCN) [6], European Society for Medical Oncology (ESMO) [7], and other thyroid associations in China, Korea [8]*, etc.,* lobectomy was the preferred surgical approach. AS was a selective choice for low-risk PTMC patients with good compliance, especially after the report of approximately 30-year follow-up results of PTMC patients who underwent AS in Kuma Hospital [9] and recommendations by Japanese guidelines. In addition, some researchers [10, 11] attempted to complete radical treatment of PTMC by RFA and obtained acceptable results.

On one hand, patients in specific occupations requiring frequent use of the voice, such as teachers, singers, moderators, commentators, worry about the progression of tumor, and on the other hand, the vocal fold paralysis (VFP) will seriously affect their careers. Moreover, young female patients with reproductive needs usually cannot accept and worry about hypothyroidism after lobectomy or total thyroidectomy (TT) [12]. Thus, for low-risk patients with specific needs, we conducted modified partial thyroidectomy, also called conformal thyroidectomy, and obtained better short-term outcomes and similar long-term oncological outcomes compared with lobectomy. Following the transformation from total thyroidectomy to lobectomy, we hope that this study will provide evidence for the feasibility of conformal thyroidectomy and contribute to minimally invasive thyroid surgery and functional protection.

## Methods

### Definition

The definition of conformal thyroidectomy was as follows: (1) preoperative ultrasound (and intraoperative ultrasound) located the peripheral margin of tumor; (2) it was ensured that the margin was more than 5 mm from the tumor; (3) intraoperative freeze biopsy proved that the margin was negative.

### Study design and patients

This retrospective study was conducted at Peking University People's Hospital, a single tertiary medical center, from 2010 to 2015. All patients were fully informed about the extent, benefits, and risks of the surgery and signed the informed consent form. The Institutional Review Board of Peking University People's Hospital provided ethical approval (2023PHB289-001) and approved the data collection and subsequent analyses in accordance with the Declaration of Helsinki, as revised in 2013. The study adheres to the STROCSS 2021 Guideline [13].

The inclusion criteria were as follows: (1) fine-needle aspiration (FNA) biopsy result was papillary thyroid cancer; (2) the stage was cT1aN0M0 according to 8th edition American Joint Committee on Cancer Staging (AJCC) [14]; and (3) there were no other severe disease or malignant tumors that threatened life.

The exclusion criteria were as follows: (1) bilateral thyroid cancer; (2) prior radiation exposure of the head and neck; (3) poorly differentiated and other poor prognosis subtypes; and (4) tumors located in specific sites for which it was difficult to ensure margins, such as the entry point of the recurrent laryngeal nerve (RLN), or other invasive prognostic factors [15, 16].

The patients who underwent conformal thyroidectomy were enrolled in the observation group, and patients who underwent lobectomy without lymph node dissection were enrolled in the control group.

### Management of follow-up

Regardless of their surgical extent, all patients were followed with physical examinations, thyroid function tests, and neck ultrasonography every 3 months. All considerations of recurrence and metastasis by ultrasound were checked by a senior radiologist, and controversy was judged by the third radiologist and surgeon who conducted the surgery. Neck and chest computed tomography with contrast, FNA, and whole-body fluorodeoxyglucose positron emission tomography were recommended if the patients considered recurrence of metastasis.

### Primary outcome

The primary outcome of this study was recurrence and metastasis, defined as new lesions after surgery confirmed by cytological or histopathological examination and/or the appearance of distant metastatic lesions on imaging studies. Event-free survival (EFS), the interval from initial surgery to the detection of recurrent or metastatic disease, was compared between groups formed according to the surgical extent.

### Statistical analysis

R 4.3.1 (R Foundation for Statistical Computing, Vienna, Austria; www.Rroject.org) and SPSS 29.0 (IBM, Armonk, NY, United States) were used for data analysis. Categorical variables were analyzed using Pearson’s χ2 test or Fisher’s exact test, according to the expected values. The Mann–Whitney U test was utilized to compare continuous variables, which are presented as medians and interquartile ranges and as the mean ± SD. The Kaplan–Meier method and log-rank test were performed to conduct survival analyses and evaluate differences in survival time, respectively. Univariate and multivariate analyses were performed using the Cox proportional hazards model. Univariate analysis was primarily performed, and variables with *P* < 0.2 were subsequently input into the multivariate analysis to determine the independent prognostic factors. Hazard ratios with their 95% confidence intervals were also derived. Statistical significance was defined as *P* < 0.05. EFS curves were constructed using the Kaplan–Meier method, and the log-rank test was used to compare EFS.

## Results

### Baseline clinical characteristics

As shown in Table 1, a total of 124 of 319 patients were enrolled in the observation group, and 195 patients were enrolled in the control group according to the surgical extent. The mean age of the patients was 49.52(± 12.50) and 47.78(± 13.10) years old in the two groups, and female patients comprised the majority (77.42% and 78.46%, respectively). Regarding tumor site and size, lesions had a mean diameter of 0.62(± 0.28) and 0.62(± 0.28) cm, and most of them were located equally in the left and right lobes in the two groups, except for six located in the isthmus (two in the observation group and four in the control group). There was no significant difference between the observation and control groups in the above four items.

**Table 1:** Baseline characteristics of total patients

	Observation Group (*n* = 124)	Control Group (*n* = 195)	*p-Value*
Age (years)	49.52(± 12.50)	47.78(± 13.10)	0.239
Gender			0.936
Female	96	153	
Male	28	42	
Tumor Site			0.881
Left lobe	63	94	
Right lobe	59	97	
Isthmus	2	4	
Tumor Size (cm)	0.62(± 0.28)	0.62(± 0.29)	0.958

### Perioperative characteristics

Following Table 2, the differences in hospital stays (0.61 days, 6.48(± 2.24) in observation group and 7.09(± 2.11) in control group), operative time (50.8 min, 42.15(± 9.35) in observation group and 92.95(± 46.35) in control group), and short-term complications (4.03%, 1.61% in observation group and 6.67% in control group) were statistically significant between the two groups. Regarding the mean bleeding volume, the loss in the observation group was 2.34 mL less than that in the control group, with no statistical significance. Interestingly, patients who underwent conformal thyroidectomy had fewer short-term complications, and only two cases of postoperative bleeding were observed. In the control group, although most patients had no long-term complications (after 10 years of follow-up, two patients suffered from persistent vocal cord paralysis, and no other long-term complications were observed), approximately 5% of them suffered from thyroidectomy-related complications and other perioperative complications (three patients were observed, one for pneumonia and two for cardiocerebrovascular accident). In addition, after 5 years of thyroid hormone replacement treatment, three patients suffered from permanent hypothyroidism in the lobectomy group, and no patients were observed in the conformal thyroidectomy group.

**Table 2:** Perioperative characteristics of total patients

	Observation Group (*n* = 124)	Control Group (*n* = 195)	*p-Value*
Hospital stays (days)	6.48(± 2.24)	7.09(± 2.11)	0.015
Operative time (minutes)	42.15(± 9.35)	92.95(± 46.35)	< 0.001
Bleeding volume (mL)	17.30(± 14.94)	19.64(± 15.23)	0.178
Short-term complications	2 (1.61%)	13 (6.67%)	0.038
Bleeding	2	1	
Hypoparathyroidism	0	4	
Vocal cord paralysis	0	5	
Others	0	3	
Permanent hypothyroidism	0	3	0.428

### Follow-up results

After ten years of follow-up(119.17(± 24.58) month in observation group and 120.10(± 25.74) in control group), all the patients survived, and nine in the observation group and 16 in the control group had recurrence or metastasis (Table 3).

**Table 3:** Follow-up results of total patients

	Observation Group (*n* = 124)	Control Group (*n* = 195)	*p-Value*
Follow-up times (months)	119.17(± 24.58)	120.10(± 25.74)	0.775
Outcome	9	16	
Lobe Recurrence	1	1	
Ipsilateral	0	0	
Contralateral	1	1	
Bilateral	0	0	
Lymph nodes metastasis	9	15	
Central neck region	4	5	
Ipsilateral	4	4	
Contralateral	0	0	
Bilateral	0	1	
Lateral cervical region	5	10	
Ipsilateral	4	6	
Contralateral	0	1	
Bilateral	1	3	

**Fig. 1:**
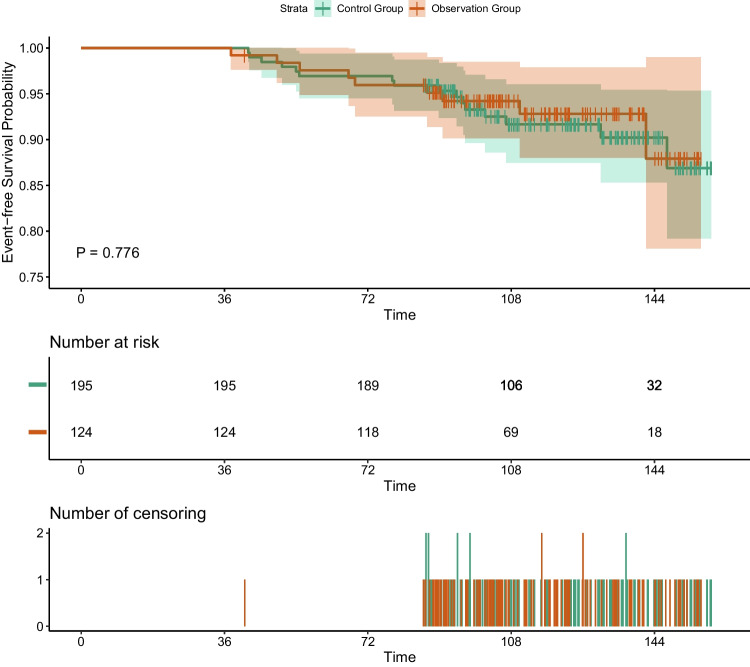
Event-free Survival Probability of Observation Group and Control Group

In patients who underwent conformal thyroidectomy, one patient had a new lesion in the contralateral lobe and ipsilateral lateral cervical lymph node, four patients had central neck lymph node metastasis, and four patients had cervical lymph node metastasis. In patients with lobectomy, one patient had a new lesion in the contralateral lobe, five patients had central neck lymph node metastasis, and ten patients had lateral lymph node metastasis. Another notable observation is that a total of five patients (one in the observation group and four in the control group) showed invasive behavior, evidenced by bilateral lymph node metastases. The 5- and 10-year EFS rates were 92.8% and 87.9% in the observation group and 93.3% and 91.7% in the control group, respectively, and there was no statistical significance between the two groups (*p* = 0.776, Fig. 1).

## Discussion

With increasing published evidence, most guidelines have demonstrated that lobectomy, instead of radioactive iodine (RAI) and TT, should become the first-line treatment for low-risk PTMC. A lower risk of complications, less hormone replacement, and an acceptable recurrence rate facilitated the acceptance of lobectomy [8, 17].

In other words, according to published records, the incidence of thyroidectomy-related lawsuits in America was 0.059% [18]. Among them, except for death due to hematoma, long-term complications such as nerve injury, especially recurrent laryngeal nerve (RLN), and hypoparathyroidism were the primary causes [19, 20]. Complications of TT and lobectomy constituted the notable percentage of 66.40%, and only a small fraction comprised by less invasive surgical procedures such as subtotal thyroidectomy and partial thyroidectomy [20].

For thyroid cancer, which had longer survival than other cancers, it seems that complications rather than recurrence, metastasis, and tumor-related death affected patient satisfaction to some degree. Thus, for patients with the specific needs mentioned above, a less invasive surgical approach instead of lobectomy was feasible and met their needs.

Based upon the same baseline characteristics with no significant difference, conformal thyroidectomy showed advantages in hospital stays, operative time, and short-term complications. According to published records, shorter hospital stays are usually associated with higher patient satisfaction and lower inpatient expenses [21]. A shorter operative time could enhance recovery after surgery and reduce anesthesia and circulatory complications. Compared with published studies [22], the complication rate in our center was acceptable; even better, postoperative bleeding was observed in two patients in the conformal thyroidectomy group and one patient in the control group, and hypoparathyroidism and VFP only occurred in the control group. Because the posterior structure of the thyroid was not exposed, conformal thyroidectomy demonstrated desirable protection of the RLN and parathyroids. In addition, consistent with the published literature [23], three patients with permanent hypothyroidism were observed in the control group, and conformal thyroidectomy reduced the rate of hypothyroidism.

Compared with previous studies [24], there was no significant difference in long-term follow-up results (10-year EFS in patients who underwent lobectomy was 92% from the literature vs. 91.7% from our studies). Although there were no criteria for the surgical margin in thyroidectomy and some studies [25] demonstrated that a positive margin was not an independent risk factor, we still chose 5 mm as the safe margin, and our study proved that radical resection with a safe margin was as safe as lobectomy. Although most PTMCs carry an excellent prognosis, some lesions display aggressive behavior (central neck, lateral cervical lymph node metastasis or distant metastasis) and fatal outcomes [26, 27]. Concerning relapsed patients, LN metastasis made up the majority, especially ipsilateral central neck and lateral cervical LNs. Interestingly, the recurrence pattern was different from that in previous studies [28], and other possible reasons, such as preoperative micrometastasis, need to be clarified. Moreover, conformal thyroidectomy gave us a glimpse at a novel surgical approach and the definition of a safe margin in thyroid cancer.

One decade ago, TT was the standard surgical approach, and conformal thyroidectomy was just an experimental surgical approach for patients with specific needs. Thus, the study was retrospective design, and the patients were not enrolled randomly. Fortunately, there were no differences between the groups in baseline characteristics, and no steps needed to be taken (such as propensity score matching) to eliminate them.

## Conclusions

As a novel treatment strategy, conformal thyroidectomy had an EFS that was not inferior to that of lobectomy but showed advantages in perioperative management and short-term complication rates. Compared with RFA, conformal thyroidectomy seems not to offer any advantages in perioperative characteristics or short- or long-term outcomes. However, the shorter learning curve and lower learning barrier of conformal thyroidectomy make it suitable for promotion in countries/areas with no developed healthcare services.

### Supplementary information

Below is the link to the electronic supplementary material.

Supplementary file1 (DOCX 29 KB)

## Data Availability

All data generated or analyzed during this study are included in this published article. Further information can be obtained from the corresponding author on reasonable request.
